# Statistical optimization of process parameters for the production of tannase by *Aspergillus flavus* under submerged fermentation

**DOI:** 10.1007/s13205-013-0139-z

**Published:** 2013-05-25

**Authors:** S. K. Mohan, T. Viruthagiri, C. Arunkumar

**Affiliations:** Department of Chemical Engineering, Annamalai University, Annamalainagar, 608002 Tamilnadu India

**Keywords:** Tannase, Response surface methodology, Plackett–Burman design, Submerged fermentation

## Abstract

Production of tannase by *Aspergillus flavus* (MTCC 3783) using tamarind seed powder as substrate was studied in submerged fermentation. Plackett–Burman design was applied for the screening of 12 medium nutrients. From the results, the significant nutrients were identified as tannic acid, magnesium sulfate, ferrous sulfate and ammonium sulfate. Further the optimization of process parameters was carried out using response surface methodology (RSM). RSM has been applied for designing of experiments to evaluate the interactive effects through a full 31 factorial design. The optimum conditions were tannic acid concentration, 3.22 %; fermentation period, 96 h; temperature, 35.1 °C; and pH 5.4. Higher value of the regression coefficient (*R*^2^ = 0.9638) indicates excellent evaluation of experimental data by second-order polynomial regression model. The RSM revealed that a maximum tannase production of 139.3 U/ml was obtained at the optimum conditions.

## Introduction

Tannin acyl hydrolase (EC 3.1.1.20), commonly known as tannase, catalyses the hydrolysis of ester and depside bonds in hydrolysable tannins such as tannic acid, resulting glucose and gallic acid (Lekha and Lonsane 1997). Tannase is extensively used in the preparation of instant tea, beer, wine, coffee flavored soft drinks, etc. Tannase is also used in the production of gallic acid, a substrate for chemical synthesis of propyl gallate and trimethoprim which have application in food and pharmaceutical industries (Das Mohapatra et al. 2006; Lekha and Lonsane 1994). Many fungi, such as *Aspergillii, Penicillii, Fusaria,* and *Trichoderma* (Mohan et al. 2012; Batra and Saxena 2005; Murugan et al. 2007; Paranthaman et al. 2009), yeast like *Candida* sp., and *Saccharomyces cerevisiae* (Mondal et al. 2001a) have been reported as tannase producers. Few bacteria like *Bacilli*, *Corynebacterium* sp., *Lactobacillus* sp., and *Serratia* sp. are known to produce tannase (Rodríguez et al. 2008; Milva et al. 2010).

In industrial level tannase is mainly produced by *Aspergillus* species under submerged fermentation (SmF). The SmF is widely used for enzyme production because it offers many advantages like uniform process conditions, viz. concentration, temperature, pH, aeration and agitation in the bioreactors (Lekha and Lonsane 1997). In addition, the utilization of agro-industrial wastes, on one hand, provides alternative substrates and, on the other hand, helps to solve pollution problems by eliminating the need for disposal of the wastes. The nature of the substrate employed is the most important factor affecting fermentation processes, and its selection depends upon several factors mainly related to cost and availability and, thus, may necessitate the screening of several agro-industrial residues (Couto and Sanroman 2006).

The use of a sequential experimental design strategy is a useful tool for process optimization. Response surface methodology (RSM) provides important information regarding the optimum level of each variable along with its interactions with other variables and their effects on product yield. It reduces the number of experiments without neglecting the interactions among the parameters. This multivariate approach also improves statistical interpretation possibilities and evaluates the relative significance of several contributing factors even in the presence of complex interactions (Dilipkumar et al. 2011). RSM is widely used for multivariable optimization studies in several biotechnological processes such as the optimization of media, process conditions, hydrogen production, fermentation, sorption of dyes, etc. (Mannan et al. 2007; Pan et al. 2008). In the present work, optimization of tannase production by *Aspergillus flavus* sp. using tamarind seed powder as substrate was carried out using a sequential strategy of the experimental design. So far no work is available on the production of tannase using tamarind seed as substrate. Tamarind seed powder is a cheap substrate which is abundantly available in southern part of India. Hence, it was selected as substrate for the production of tannase.

## Materials and methods

### Microorganism and culture conditions

The tannase producing fungal culture, *A. flavus* (MTCC 3783) was obtained from IMTECH, Chandigarh and used for tannase production. The fungal culture was maintained on Czapek Dox minimal media agar slants supplemented with 1 % tannic acid as the sole carbon source. The fungal strain was sub-cultured periodically, grown at 30 °C for 7 days. The well grown culture was stored at 4 °C in a refrigerator and used for further sub-culturing.

The composition of the Czapek Dox minimal medium used for tannase enzyme production was: tannic acid, 10 g/l; sodium nitrate, 6 g/l; potassium dihydrogen orthophosphate, 1.52 g/l; magnesium sulfate, 0.52 g/l; potassium chloride, 0.52 g/l; ferrous sulfate, 0.01 g/l and zinc sulfate, 0.01 g/l. Cells were harvested from slants and used to inoculate liquid medium.

### Production of tannase in submerged fermentation (SmF)

The spore suspension was inoculated in 250 ml Erlenmeyer flask containing 100 ml of Czapek Dox minimal medium. 3 gm of substrate (tamarind seed) was added separately to the above mentioned medium for studying their effect on the enzyme production. The cultures were grown at 30 °C, 140 rpm for 6 days in an incubator shaker. The samples were withdrawn at regular intervals of 24 h. The biomass was separated by filtering through Whatman No.1 filter paper. The cell free culture broth was assayed for the tannase activity.

### Assay of tannase

0.1 ml of enzyme solution was incubated with 0.3 ml of 1.0 % (w/v) tannic acid and 0.2 M acetate buffer (pH 5.0) at 40 °C for 10 min and then the enzyme production was stopped by cooling to 0 °C by the addition of 2 ml bovine serum albumin (BSA) (1 mg/ml), which precipitates the remaining tannic acid. Simultaneously, a control without the enzyme was incubated and the samples were analyzed. The tubes were then centrifuged (5,000×*g*, 10 min) and the precipitate was dissolved in 2 ml of sodium dodecyl sulfate (SDS)—triethanolamine (1 % w/v SDS in 5 % v/v triethanolamine) solution and the absorbency was measured at 550 nm after addition of 1 ml of FeCl_3_ (0.01 M FeCl_3_ in 0.01 N HCl) (Mondal et al. 2001b). One unit of tannase enzyme was defined as the amount of enzyme required to hydrolyze one micro mole of ester linkage of tannic acid in 1 min at specific condition.

### Plackett–Burman design

Plackett–Burman design, an efficient technique for medium component selection (Plackett–Burman 1946) was used to determine the factors that significantly influence the tannase production. Twelve variables (Table 1) were screened in 20 experimental runs (Table 2) and the insignificant variables were eliminated to obtain a smaller, more manageable set of factors. The low level (−1) and high level (+1) of each factor are listed in Table 1. The statistical software package ‘Design Expert 7.1.5’ was used for analyzing the experimental data.

**Table 1:** Nutrient screening using a Plackett–Burman design

Levels (g/ml)			
Nutrient code	Nutrient	Low (−1)	High (+1)
A	Tannic acid	1	5
B	Yeast extract	0.1	1
C	Magnesium sulfate	0.1	1
D	Ferrous sulfate	0.1	1
E	Ammonium nitrate	0.1	1
F	Ammonium chloride	0.1	1
G	Urea	0.1	1
H	Potassium chloride	0.1	1
I	Sodium nitrate	0.1	1
J	Potassium dihydrogen phosphate	0.1	1
K	Ammonium sulfate	0.1	1
L	Peptone	0.1	1

**Table 2:** Plackett–Burman experimental design matrix for screening of important variables for tannase production using *Aspergillus flavus*

Run no.	*A*	*B*	*C*	*D*	*E*	*F*	*G*	*H*	*I*	*J*	*K*	*L*	Tannase (U/ml)
1	1	1	1	1	−1	−1	1	1	−1	1	1	−1	38.20
2	−1	1	−1	1	−1	1	1	1	1	−1	−1	1	65.40
3	−1	−1	−1	−1	1	−1	1	1	1	1	−1	−1	95.52
4	−1	1	1	−1	−1	−1	−1	1	−1	1	−1	1	80.82
5	1	−1	1	−1	1	1	1	1	−1	−1	1	1	39.67
6	1	−1	−1	−1	−1	1	−1	1	−1	1	1	1	58.80
7	−1	−1	−1	1	−1	1	−1	1	1	1	1	−1	42.80
8	1	−1	1	1	−1	−1	−1	−1	1	−1	1	−1	30.87
9	−1	1	−1	−1	1	−1	−1	−1	−1	−1	1	−1	50.89
10	−1	1	−1	−1	1	1	−1	1	1	−1	−1	−1	88.76
11	−1	−1	−1	−1	−1	−1	−1	−1	−1	−1	−1	−1	90.76
12	−1	1	1	1	1	−1	−1	1	1	−1	1	1	43.90
13	−1	−1	1	1	−1	1	1	−1	−1	−1	−1	1	57.90
14	−1	−1	−1	−1	1	−1	1	−1	1	1	1	1	86.54
15	1	−1	1	1	1	1	−1	−1	1	1	−1	1	38.00
16	−1	1	−1	1	1	1	1	−1	−1	1	1	−1	54.89
17	1	1	1	−1	−1	1	1	−1	1	1	−1	−1	47.02
18	1	1	−1	−1	−1	−1	1	−1	1	−1	1	1	60.76
19	1	−1	−1	1	1	−1	1	1	−1	−1	−1	−1	56.76
20	1	1	−1	1	1	−1	−1	−1	−1	1	−1	1	69.00

### Box-Behnken design

The process variables with significant effects on enzyme production were optimized using Box-Behnken design. To determine the response pattern and synergy of variables the full 2^*k*^ composite design was performed giving 2^*k*^ + 2*k* + *n*_0_ combinations where *k* is the number of independent variables and n_0_ is the number of replications of the experiments at center point. This provided 31 experimental runs performed with four factors at five coded levels (−2, −1, 0, +1 and +2) in duplicate, with central points in triplicate to determine the experimental error (Box and Draper 1987). The coded and actual values of the variables are presented in Table 3. The responses of the input variables were evaluated as a function of tannase activity, measured as the amount of tannase liberated and coded by Y (U/ml) (Table 4). A multiple regression analysis of the data was carried out to obtain an empirical model that relates the measured response to the independent variables. A second-order polynomial equation was obtained.

**Table 3:** Ranges of the independent variables used in RSM

Variables	Levels (U/ml)					
	Code	−2	−1	0	+1	+2
Tannic acid conc. (%)	*X*_1_	1	2	3	4	5
Fermentation period (h)	*X*_2_	48	72	96	120	144
Temperature (^o^C)	*X*_3_	25	30	35	40	45
pH	*X*_4_	4.5	5	5.5	6	6.5

**Table 4:** Central composite design (CCD) of factors in coded levels with enzyme activity as response

Run no.	*X*_1_	*X*_2_	*X*_3_	*X*_4_	Tannase activity (U/ml)	
					Experimental	Predicted
1	0	0	2	0	100.45	95.98
2	0	0	−2	0	94.6	90.37
3	0	0	0	0	137.54	134.71
4	0	−2	0	0	98.5	94.96
5	−1	1	−1	1	78.5	81.94
6	−1	−1	−1	1	85.5	84.56
7	1	1	−1	1	90.67	94.55
8	1	1	1	−1	95.67	98.75
9	0	0	0	2	90.54	85.72
10	−1	1	−1	−1	99.54	99.19
11	0	0	0	0	134.32	134.71
12	−1	−1	1	−1	100.43	98.91
13	1	−1	1	−1	95.7	98.61
14	0	0	0	0	134.23	134.71
15	0	0	0	0	134.13	134.71
16	1	−1	−1	1	95.34	97.73
17	0	0	0	0	134.22	92.49
18	0	0	0	0	134.31	134.71
19	1	1	1	1	97.43	97.73
20	0	2	0	0	97.65	92.49
21	1	1	−1	−1	103.21	105.65
22	0	0	0	0	134.23	134.71
23	−1	1	1	1	90.36	92.72
24	−1	−1	1	1	93.15	97.06
25	−1	1	1	−1	96.22	99.90
26	−1	−1	−1	−1	90.43	96.48
27	−2	0	0	0	106.4	102.44
28	1	−1	1	1	100.2	102.913
29	0	0	0	−2	102.56	98.67
30	2	0	0	0	119.5	114.75
31	1	1	1	−1	98.4	98.76

1where *Y* is the predicted response, β_0_ is the intercept, β_*i*_ is the linear coefficient, β_*ii*_ is the quadratic coefficient and β_*ij*_ is the interaction coefficient. The relation between the coded forms of the input variable and the actual values of chosen variables is described as follows:2where *x*_*i*_ is the coded value, *X*_*i*_ is the actual value of an independent variable, *X*_0_ is the value of *X*_*i*_ at center point and Δ*X*_*i*_ is the step change of the variable and *i* = 1–4. The analysis of results was performed with statistical and graphical analysis software (Design expert 7.1.5). The software was used for regression analysis of data obtained and to estimate the coefficient of regression equation. ANOVA (analysis of variance) for the final predictive equation and optimization values were also obtained using the same software package which indicates whether the variable was more significant or less significant.

## Results and discussion

### Screening of nutrients by PBD

**Fig. 1:**
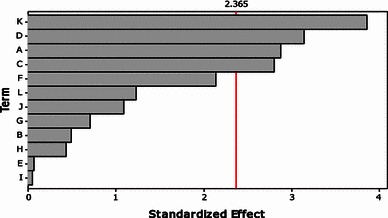
Pareto chart showing the effect of media components on tannase activity using *Aspergillus flavus*

Experiments were carried out based on Plackett–Burman design and the results obtained were given in Table 2. From the table, it was observed that the variation in tannase activity was 30–90 U/ml. From the Pareto chart (Fig. 1), the nutrients, tannic acid, magnesium sulfate, ferrous sulfate and potassium dihydrogen phosphate were found to be significant for the production of tannase by *A. flavus* sp. using tamarind seed powder as substrate.

The equation obtained waswhere A, tannic acid; B, yeast extract; C, magnesium sulfate; D, ferrous sulfate; E, ammonium nitrate; F, ammonium chloride; G, urea; H, potassium chloride; I, sodium nitrate; J, potassium dihydrogen phosphate; K, ammonium sulfate; L, peptone.

From the above equation, the negative coefficient for K, D, A and C indicates that increase in the nutrient concentration decreases the tannase activity. Hence their levels were maintained at their low (−1) levels in the optimization of process variables.

### Optimization of process parameters by RSM

The levels of the factors (tannic acid concentration, fermentation period, temperature and pH) and the effect of their interactions on tannase production were determined by central composite design of RSM. Thirty-one experiments were performed with different combinations of the factors shown in Table 4. The predicted and observed responses along with the design matrix were presented in Table 5. The results were analyzed by ANOVA. The second-order regression equation provided levels of tannase activity as a function of tannic acid concentration, fermentation period, temperature and pH which can be presented in terms of coded factors as in the following equation:

**Table 5:** Analysis of variance (ANOVA) for response surface quadratic model for the production of tannase

Source	Coefficient factor	Sum of squares	*DF*	*F*	*P* > *F*
Model	134.79	8,045.48	14	28.49	<0.0001
*X*_1_	3.08	209.76	1	10.40	0.0057
*X*_2_	−0.62	8.64	1	0.43	0.5228
*X*_3_	1.40	44.48	1	2.21	0.1582
*X*_4_	−3.24	231.90	1	11.50	0.0040
*X*_1_ * × X*_2_	−0.21	0.65	1	0.032	0.8604
*X*_1_ * × X*_3_	−1.90	52.81	1	2.62	0.1265
*X*_1_ * × X*_4_	1.54	33.68	1	1.67	0.2160
*X*_2_ * × X*_3_	−0.43	2.64	1	0.13	0.7227
*X*_2_ * × X*_4_	−1.33	25.97	1	1.29	0.2742
*X*_3_ * × X*_4_	2.52	92.71	1	4.60	0.0483
*X*_1_ * × X*_1_	−6.55	1,169.38	1	57.98	<0.0001
*X*_2_ * × X*_2_	−10.27	2,874.51	1	142.53	<0.0001
*X*_3_ * × X*_3_	−10.41	2,952.02	1	146.37	<0.0001
*X*_4_ * × X*_4_	−10.65	3,091.94	1	153.31	<0.0001
Residual	–	302.52	15	–	–
Lack of fit	–	289.31	9	14.60	0.0020
Pure error	–	13.21	6	–	–
Cor total	–	8,348.00	29	–	–

Std. Dev. 4.49; *R*^2^ 0.9638; Mean 104.33; Adj *R*^2^ 0.9299; CV % 4.30; Pred *R*^2^ 0.7785; Adeq Precision 16.644

 where *Y* is the tannase activity (U/ml) produced as a function of the coded levels of tannic acid concentration (*X*_1_), fermentation period (*X*_2_), temperature (*X*_3_) and pH (*X*_4_).

The ANOVA for tannase production was given in Table 5. A Model F value of 28.49 implies the model was significant. Values of “Prob>F” <0.05 indicate the model terms were significant. Values >0.1 indicate the model terms were not significant. In the present work, the linear effect of tannic acid concentration and pH, square effects of *X*_1_, *X*_2_, *X*_3_ and *X*_4_ and interactive effects of *X*_3_ and *X*_4_ were significant for tannase production. The coefficient of determination (*R*^2^) for the tannase activity was calculated as 0.9638, which is close to 1 and can account for up to 96.38 % of the variability of the response. The predicted *R*^2^ value of 0.7785 is in reasonable agreement with the adjusted *R*^2^ value of 0.9299. An adequate precision value >4 is desirable. The adequate precision value of 16.64 indicates an adequate signal and suggests that the model can be used to navigate the design space. The coefficient of variation (CV) indicates the degree of precision with which the experiments are compared. Generally, the higher the value of the CV is the lower the reliability of the experiment. Here a lower value of CV (4.30) indicates greater reliability of the experiments performed. The above model can be used to predict the tannase production within the limits of the experimental factors.

### Response surface plots

**Fig. 2:**
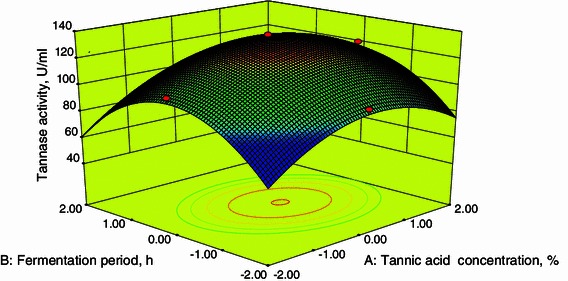
3D plot showing the effect of tannic acid concentration and fermentation period on tannase activity

**Fig. 3:**
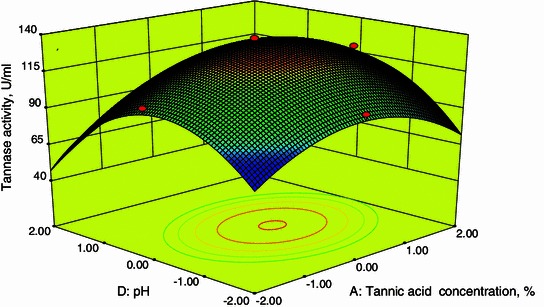
3D plot showing the effect of tannic acid concentration and pH on tannase activity

**Fig. 4:**
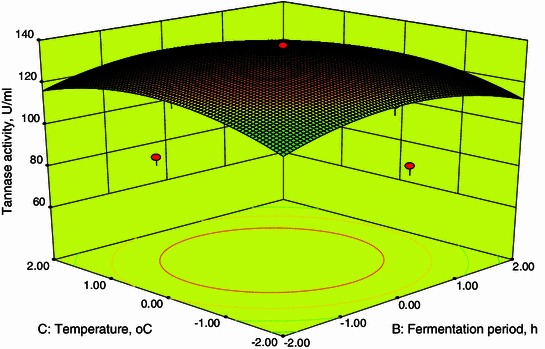
3D plot showing the effect of fermentation period and temperature on tannase activity

**Fig. 5:**
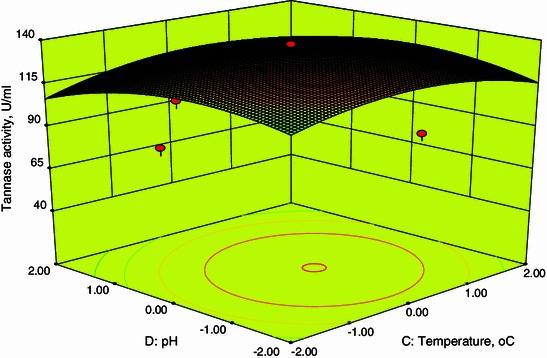
3D plot showing the effect of temperature and pH on tannase activity

The interactive effects of variables on tannase production were studied by plotting 3D surface curves against two independent variables, while keeping the other variable at its central (0) level. The 3D curves of the calculated tannase production and contour plots from the interactions between the variables were shown in Figs. 2, 3, 4 and 5. Figure 2 shows the dependency of tannase on tannic acid concentration and fermentation period. The tannase activity increased with an increase in tannic acid concentration to about 3.22 %. Tannic acid is an inducer for the production of tannase and hence tannic acid activity increases. But at higher concentration (above 3.22 %) tannase activity decreases. This may be due to inducer solubility, toxicity level and saturation which determine the optimum concentration (Pinto 2003).

From Fig. 2, it was also inferred that an increase in fermentation period resulted in an increase in tannase activity up to 96 h after that the tannase activity decreases. A decline in enzyme activity after 96 h of fermentation may be due to decrease in nutrient availability in the medium. These are in agreements with the results obtained by Beniwal and Chhokar (2010). Figures 4 and 5 show the dependency of tannase activity on temperature. Generally, temperature influences the metabolic activity of cells. At lower and higher temperatures, the tannase activity was found to be low. The maximum tannase activity was observed at a temperature of 35.1 °C. These results are well supported by Beniwal and Chhokar (2010). Similarly for pH, at low and higher levels the activity was low (Figs. 3, 5). Tannase activity was maximum in the acidic pH range, and its activity decreases in the alkaline range (Rodríguez et al. 2008; Raghuwanshi et al. 2011). The effect of pH on the activity of tannase may be attributed to its amino acids and active site which undergoes protonation or deprotonation; in addition, it may attributed to the conformational changes induced by amino acids ionization (Sabu et al. 2005).

The optimum conditions for the maximum production of tannase were determined by the response surface analyses and also estimated from the regression equation. The optimum conditions were tannic acid concentration, 3.22 %; fermentation period, 96 h; temperature, 35.1 °C; and pH 5.4. The predicted results were shown in Table 4. The predicted values from the regression equation closely agreed with the experimental values. Validation of the experimental model was tested by carrying out a batch experiment under optimal operating conditions. Three repeated experiments were performed and the results were compared. The tannase activity obtained from the experiments was very close to the response predicted by the regression model, which proves the validity of the model. At these optimized conditions the maximum tannase activity was found to be 139.3 U/ml.

## Conclusions

The response surface methodology was effectively applied for the production of tannase from *A. flavus,* MTCC 3783 using tamarind seed powder as a substrate in submerged fermentation. Central composite design was employed to evaluate the effects of tannic acid concentration, fermentation period, temperature and pH on production of tannase by *A. flavus.* Using the above optimized nutrient solution, maximum tannase activity of 139.3 U/ml was obtained at the inducer tannic acid concentration of 3.22 %, temperature of 35.1 °C, pH of 5.4, and fermentation period of 96 h. The statistical design of experiment offers efficient methodology to identify the significant variables and to optimize the factors with minimum number of experiments for tannase production by *A. flavus.*
